# Relationship between a Maximum Plank Assessment and Fitness, Health Behaviors, and Moods in Tactical Athletes: An Exploratory Study

**DOI:** 10.3390/ijerph191912832

**Published:** 2022-10-07

**Authors:** Megan Sax van der Weyden, Michael Toczko, Marcie Fyock-Martin, Joel Martin

**Affiliations:** School of Kinesiology, George Mason University, Manassas, VA 20109, USA

**Keywords:** core endurance, aerobic fitness, energy, military, body composition

## Abstract

A maximum plank hold (PH) has been implemented in the Army Combat Fitness Test (ACFT) with the Holistic Health and Fitness (H2F) program. The H2F program introduces a shift in wellbeing from a fitness centered approach to framework also comprising nutrition, sleep, mental, and spiritual components. The purpose was to analyze how a maximum PH correlated with fitness, lifestyle behaviors, and mood states in tactical athletes (TA) and assess differences between those who pass and fail. Forty-nine TA completed fitness testing, lifestyle behavior, and mood state surveys. Bivariate correlations were used to examine relationships with PH performance. PH time was significantly correlated with total body mass, fat mass, BMI, push-ups, and state physical energy (SPE). VO_2max_ was significantly different between the groups who passed and failed the PH. PH was not associated with lifestyle behaviors or trait mood states. PH performance could vary day-to-day as it was correlated with SPE. Individuals with poorer aerobic fitness and body composition may be at risk for failing the PH.

## 1. Introduction

Tactical athletes (TA) are composed of law enforcement, firefighter, and military personnel. Physical aspects of TA’s duties require muscular strength, muscular endurance, power, agility, speed, anaerobic, and aerobic fitness [1,2]. Deficiencies in any area of fitness may compromise safety, mission success, and increase risk of musculoskeletal injury. Despite a robust list of physical requirements needed to complete varying occupational tasks, fitness tests for TA have historically emphasized local muscular endurance and aerobic fitness [3,4]. Research has concluded that these fitness tests provided an incomplete assessment of TA’s occupational readiness and need to assess more areas of fitness related to job tasks that require high levels of force and power production [5]. Thus, attempts have been made to establish new fitness testing protocols to better assess all realms of fitness associated with occupational tasks [6].

The Army Combat Fitness Test (ACFT) was developed by the United States (U.S.) Army to replace the Army Physical Fitness Test (APFT) that had been in use for four decades [3]. To assess core function, the ACFT now replaces the APFT’s sit-up assessment with the plank hold (PH). The ACFT assesses soldiers against an age and gender-scaled standard [6]. Notably, the PH is now the only event graded against a gender-neutral standard. Due to scrutiny over the ACFT, a congressionally mandated independent review by the RAND Corporation was conducted to better determine the efficacy of this new occupational fitness assessment. This review found incomplete evidence to support the use of the ACFT in the Army’s soldiers and no conclusive evidence to support the leg tuck or PH event predicting performance on combat tasks or injury risk [7].

Traditionally, TA fitness assessments have utilized maximum sit-ups as a measure of local core muscular endurance [3]. As a result, the majority of previous literature has correlated only the sit-up event with performance on fitness tests and military occupational tasks [8]. Meta-analyses have shown only weak to moderate significant correlations between sit-up performance and performance on occupationally specific tasks [8]. The PH event requires isometric contraction of the total core and has been correlated with performance on tasks that are integral to TA such as marksmanship, balance, one-repetition maximum box lift, and ruck march performance [9,10,11]. Greater core strength, endurance, and stability may also reduce the risk of injuries [12]. While aspects of TA occupational duties may differ, TA present similar injuries. Military [13], law enforcement [14], and firefighters [15] all frequently report low back pain. Historically, sit-ups have been used to assess core endurance but evidence suggests that sit-ups can increase compressive forces in the lumbar spine, a contraindication for individuals with low back pain [16]. The PH is easy to implement in tactical athlete populations and has more validity than sit-ups in assessing core endurance and predicting injury risk [17]. Likewise, PH training has been shown to reduce low back pain [18].

In recent decades, a holistic approach to wellness has been adopted by the military, fire, and police departments. Concomitantly with the ACFT, the U.S. Army is implementing the Holistic Health and Fitness (H2F) program. The H2F program represents a paradigm shift from physical fitness being viewed as the primary determinant of solder readiness to holistic framework comprising nutrition, sleep, mental, and spiritual, along with physical, components. Law enforcement agencies [19] and firefighter departments [20] have also begun adopting more holistic views of tactical athlete health. Moving forward, in addition to promoting physical fitness, there is an emphasis on healthy lifestyle behaviors and mental health [21,22]. The “Big 3” modifiable healthy lifestyle behaviors of sleep, physical activity, and diet have been positively correlated with mental health and well-being in adults [23]. Unfortunately, the shiftwork and unpredictable hours of TA’s occupations often lead to sleep deprivation, long hours of sitting, and poor diets [24,25]. Poor lifestyle behaviors can, in turn, have a negative effect on TA’s mental and emotional health and ultimately, performance and longevity.

Worsened mental health (i.e., depression) can present as feelings of lower energy and higher fatigue. State and trait mental and physical energy and fatigue may influence physical performance and have been shown to be correlated with balance which could influence injury risk [26]. Negative mood states have been associated with decreased performance on a ruck march in the summer at U.S. Army Ranger School [27]. Interestingly, certain personality types may be less likely to adopt unhealthy lifestyle behaviors. For example, the personality trait known as grit (i.e., the inclination to pursue long-term goals with sustained interest and effort over time) appears to have a positive influence on living a healthy lifestyle in terms of the “Big 3” modifiable behaviors [28]. Moreover, grit has been reported to be associated with better physical performance in U.S. Military Academy cadets and increased retention in a U.S. Army Special Operations Forces selection course [29,30]. Thus, state and trait energy and fatigue, grit, and mood states may be factors influencing operational readiness in TA.

The primary aim of this study was to examine whether the maximum PH performance is associated with common fitness assessments, modifiable lifestyle behaviors (i.e., sleep, diet, and physical activity), as well as moods and personality in TAs. We hypothesized that PH performance would be positively associated with body composition, aerobic endurance, and upper body muscular endurance as well as healthy lifestyle behaviors and grit. Given the recent adoption of the maximal PH assessment on the ACFT and gender-neutral scoring, there is some uncertainty of the implications of the pre-determined cut-off time to determine passing, or failing, of the PH. Thus, a secondary aim was to assess differences in fitness, lifestyle, and health between those who pass and fail the PH event based on ACFT standards. We hypothesized that those who passed the PH event would be fitter and report living a healthier lifestyle. Our analyses utilized an approach similar to current holistic frameworks of performance optimization employed by a majority of TA organizations.

## 2. Materials and Methods

### 2.1. Experimental Approach to the Problem

A cross-sectional observational design was used in which participants were required to complete a standardized testing battery. Testing was completed during a single 90-min session and each participant was tested individually. Participants were asked to avoid strenuous exercise up to 48 h before testing and avoid food or fluid intake other than water for 2 h before testing. Upon reporting to the laboratory, participants completed an informed consent followed by a series of electronic questionnaires regarding moods, personality, and lifestyle behaviors. Once the questionnaires were completed, anthropometric measures (i.e., height, mass, and body composition) were obtained. Participants then performed a series of movement assessments (i.e., wall sit and reach, Y-balance test, overhead squat, and Apley scratch test). Next, a dynamic warm-up was performed before completing the following fitness assessments in this order: countermovement jump, 1-repetition maximum bench press, pull-up, push-up, PH, and maximal oxygen consumption test. All participants were provided familiarization and standard instructions for the assessments. All test sessions were conducted by researchers at George Mason University. For all testing sessions, at least one of the researchers present was a National Strength and Conditioning Association Certified Strength and Conditioning Specialist.

### 2.2. Subjects

The sample of the study consisted of 49 (males = 41, females = 8) tactical athletes (law enforcement officers, *n* = 29; firefighters, *n* = 20) 38 ± 7.8 years of age with 12 ± 8.3 years of service. Participation in the study was purely voluntary and there were no formal requirements to participate as part of their employment. Participants were recruited via email and flyers. To be eligible for the intervention, participants were required to: (1) be either recruits or currently employed emergency responders in Northern Virginia; (2) not have surgery or injury in the last 3 months; (3) ability to run, perform pull-ups, and push-ups without pain; (4) no history of cardiovascular, pulmonary, renal, or metabolic disease; and (5) engage on average 30 min of physical activity daily. All participants were informed of the benefits and risks of the study and signed the informed consent. The study was approved by George Mason University’s Institutional Review Board (IRB #: 12179B). Data was collected using appropriate safeguards to protect participant’s identifying information. All procedures were conducted in accordance with best practices related to ethical issues in exercise science research [31].

### 2.3. Procedures

The order of testing was the same for all participants. Upon arrival to the testing facility, participants completed the informed consent and questionnaires. The anthropometric measurements were then taken. The movement and fitness assessments were performed afterwards. To minimize the effect of fatigue, each participant was given exactly 5 min of rest between fitness assessments. Participants performed a dynamic warm-up using a standard and supervised procedure to minimize risk of skeletal muscle injury during fitness assessments. Rest periods were derived from pilot testing of the protocol.

### 2.4. Questionnaires

Participants completed a series of electronic questionnaires to assess lifestyle behaviors, personality, and mood states. The order in which the questionnaires were completed was as follows.

#### 2.4.1. Energy and Fatigue

The mental and physical energy and fatigue scale was used to measure the complexity of mental energy, mental fatigue, physical energy, and physical fatigue. The validity and reliability of this instrument has been supported by the work of Boolani et al. [32] and O’Connor [33]. State responses were assessed on a 100-point scale from “never” to “always”. Trait responses were assessed on 10-point scale from “never” to “always”. Participants completed mood state questionnaires prior to and following 90-min of strenuous exercise. The Cronbach’s alpha for trait and state mood were as follows: trait physical energy = 0.92, trait physical fatigue = 0.87, trait mental energy = 0.82, trait mental fatigue = 0.91, state physical energy = 0.78, state physical fatigue = 0.91, state mental energy = 0.73, and state mental fatigue = 0.94.

#### 2.4.2. Grit

The 8-item short grit scale (Grit-S) was used to measure grit, defined as perseverance and passion towards long-term goals [34]. An aggregated score was divided by 8 to determine total grit scores ranging from 1 (not at all gritty) to 5 (extremely gritty). The Cronbach’s alpha for the current study was 0.76.

#### 2.4.3. Diet

Dietary behaviors were assessed via the Rapid Eating Assessment for Participants Short Version (REAP-S), a 16-item questionnaire including 13 items addressing first part frequency of food choices (i.e., In an average week, how often do you: Eat less than 2 servings of fruit a day?) and 3 items addressing the will to change dietary behaviors [35]. Higher summation of the first 13-items were indicative of healthier diets. Participants reporting <5 scores of 1 were categorized as having good diets and participants with ≥5 scores of 1 were categorized as having poor diets. The REAP-S questionnaire has good test-retest reliability and is a valid instrument when compared with the Healthy Eating Index (r = 0.472, *p* < 0.001) for measuring dietary behavior [35]. The Cronbach’s alpha for the current study was 0.71.

#### 2.4.4. Sleep

The Pittsburgh Sleep Quality Index (PSQI) was self-reported to assess sleep quality. The PSQI is a 19-item questionnaire that scores seven components: subjective sleep, sleep latency, sleep duration, sleep efficiency, sleep disturbance, use of sleep medication, and daytime dysfunction. A total sum is then reported as an overall PSQI global score. Participants were categorized as ‘good’ and ‘bad’ sleepers. Good sleep was quantified as a PSQI global score that is less than or equal to 5, while bad sleep is a PSQI global score of more than 5. Using frequency of distribution, we defined the top and bottom third of PSQI scores as extreme values of good and bad sleepers (PSQI ≤ 3 and PSQI ≥ 7). The PSQI survey has demonstrated acceptable test-retest reliability (r = 0.87), high sensitivity (98.7%), and specificity (84.4%) [36]. The Cronbach’s alpha for the PSQI for the current study was 0.73.

#### 2.4.5. Physical Activity

Participants were asked to self-report physical activity (PA) through the International Physical Activity Questionnaire-Short Form (IPAQ-SF), a 7-item scale including the frequency (exercise sessions per week), duration (minutes per session), intensity (light, moderate, vigorous), and time spent seated (hours and minutes) over the previous seven-day period. The IPAQ-SF has a moderate to high degree of reliability with Interclass Correlation Coefficients (ICC) between 0.71 and 0.89 [37,38].

### 2.5. Anthropometric Measures

Height and weight were recorded to the nearest 0.01 cm and 0.01 kg, respectively, using a stadiometer (Detecto, Webb City, MO, USA) and digital scale (BOD POD; Cosmed USA, Concord, CA, USA). Percent body fat, fat mass, and fat-free mass were measured using air displacement plethysmography (BOD POD model 2000A; BOD POD, Cosmed USA, Concord, CA, USA) following standardized procedures by the manufacturer. Air displacement plethysmography has been shown to be a reliable and valid method of assessing body composition [39].

### 2.6. Movement Assessments

#### 2.6.1. Wall Sit and Reach

The WSR test was administered to determine flexibility using the methods described in the study by Liemohn et al. [40]. Trials were measured in cm. The WSR has been previously reported to be a valid (r = 0.77) and reliable (ICC = 0.95) test to assess lumbosacral flexibility [41].

#### 2.6.2. Y-Balance Test

The YBT device (Functional Movement Systems, Chatham, VA, USA) was used to collect dynamic postural stability data following the methods described by Wright et al. in the anterior direction [42]. Performances were measured in cm. The YBT has been reported to be valid and reliable [43].

#### 2.6.3. Overhead Squat and Shoulder Mobility

To assess hip mobility, participants completed 3 repetitions of an overhead squat. The overhead squat was performed and assessed in accordance with the Functional Movement Screen™ protocol [44]. Shoulder mobility was assessed using the Apley Scratch Test. Participants were instructed to make a fist with each hand. Then, they reached one arm overhead, with their fist behind their neck, and reached the other arm to the small of their back, reaching upwards as far as they can [44]. Distance between participants’ fists was measured in cm. All steps were repeated for the measure on the other side. The functional movement screen deep squat and Apley Scratch assessments have been reported to have strong inter-rater and intra-rater reliability [45].

### 2.7. Fitness Testing

#### 2.7.1. Countermovement Jump

The countermovement VJ was used to measure lower body power. Participants performed the VJ assessment directly following a dynamic warm-up. Instructions were to use a countermovement technique and jump as high as possible on each attempt. Participants were given 2 warm-up jumps at 50% and 75% effort, respectively. Three attempts were completed, and the highest jump height was recorded. The VJ was performed on a timing mat (Just Jump, Perform Better, Cranston, RI, USA). The use of flight time to measure jump height has been reported to be valid for assessing countermovement jump performance [46].

#### 2.7.2. Upper Extremity Muscular Fitness Testing

Common upper extremity muscular fitness tests were performed to profile the upper body pushing and pulling ability of participants. A 1RMBP was used to assess upper body strength. The 1RMBP has been reported to have good to excellent test-retest reliability [47]. Posterior upper body muscular endurance was measured with pull-ups to failure. Anterior upper body muscular pushing endurance was measured with push-ups to failure. These assessments were conducted following a previously published protocol [48].

#### 2.7.3. Prone Forearm Plank

Core muscular endurance was assessed with a prone forearm PH for maximum time. Participants were required to perform the PH with forearms on the ground, elbows at 90 degrees, and contacting the ground directly below the shoulders. The head, shoulders, hips, knees, and ankles were required to be in a straight line during the test. The posture was the same as required by the ACFT PH assessment. A demonstration of the correct posture was provided prior to the start of the test. Instructions were to “keep a straight line between your shoulders, hips, knees and ankles” and “hold the position as long as possible”. The researcher began the timer when the participant initiated the PH. The timer was stopped when the participant exhibited volitional fatigue or noticeable degradation in form of the PH. At no point in the test were participants informed of the duration of the PH. Once subjects were in the correct position, the test began exactly 5 min following the push-up assessment. During the testing, subjects were provided 1 warning if they began to deviate from the correct posture. Time was recorded to the nearest second on a stopwatch. The isometric prone PH has been reported to be a valid and reliable assessment of core muscle function [49].

#### 2.7.4. Maximal Oxygen Consumption

Participants completed the Wellness-Fitness Initiative (WFI) Treadmill ramp protocol which has been validated in tactical athlete populations [50]. Peak VO_2_ was assessed; tests were terminated due to an RER ≥ 1.15, plateau, or reduction in exercising heart rate with an increase in workload, or volitional fatigue. Breath-by-breath indirect calorimetry was measured by a calibrated metabolic cart (TrueOne 2400, Parvo Medics, Salt Lake City, UT, USA). Heart rate was continuously recorded with a wearable chest strap monitor (H10, Polar-Electro, Kempele, Finland). Physiological variables were continuously monitored and recorded during all stages of the test. Exertion was self-reported at each stage of the test via the 15-point (6–20) Borg rating of perceived exertion (RPE) scale. Prior to that start of the VO_2max_, participants were shown a visual of the 15-point RPE scale and were verbally instructed that 6 was considered extremely easy (i.e., laying down watching television) and 20 was maximal effort (i.e., pushing a boulder up a mountain). In the last 30-s of each stage, the researcher held up the RPE visual and participants were verbally instructed to point to the number that corresponded with their physical efforts.

### 2.8. Statistical Analysis

The data collected, analyzed, and presented in this study are from a larger on-going project. Recently, survey questionnaires to assess personality and mood were added to the testing protocol. This led to a total sample size of 49 for anthropometric, movement, and fitness measures, but only 18 participants completing the additional measures of lifestyle, personality, and mood states. The data reported are not the primary focus of the on-going project and as a result, apriori sample size calculations were not conducted. However, apriori sample size calculations in G*Power (version 3.1.9.7, Heinrich-Heine-Universitat Dusseldorf, Dusseldorf, Germany) were computed for correlations (tails = 2, alpha = 0.05, power = 0.8) and for large effects, a sample size of 26 would be adequate. Data were compiled, cleaned, and scored into Microsoft Excel (Microsoft Inc., Redmond, WA, USA). Normality was assessed with Shapiro–Wilk test and visualized with Q-Q plots. Normality testing revealed that a PH was normally distributed, but a majority of data were not normally distributed.

Spearman Rho rank tests were run to determine correlations between PH and measures of body composition, fitness, personality, and mood states. Duration of PH was transformed into a dichotomous variable of “Pass” or “Fail” based on the cut-off times set by the U.S. Army [51]. The composition of the groups were as follows: (1) Anthropometric, movement and fitness measures: Pass group: *n* = 41, males: 35, females: 6, fire: 17, police: 34; Fail group: *n* = 8, males: 6, females:2, fire: 3, police: 5; and (2) Lifestyle, personality and mood state measures: Pass group: *n* = 16, males: 15, females: 1, fire: 1, police: 15; Fail group: *n* = 2, males: 2, females: 0, fire: 0, police: (2) Group differences between those who passed cut off times and those who did not were determined with the use of Mann–Whitney U tests. Effect sizes of Mann–Whitney U tests were determined with the Glass rank biserial coefficient (rg) and interpreted as small (rg = 0.11 to <0.28), medium (rg = 0.28 to <0.43), and large (rg ≥ 0.43) [52]. Sex differences in fitness variables were assessed, following the primary analyses, with Mann–Whitney U tests. All analyses were completed using the R Environment and packages psych, car, Rcdmr, mlogit, ggplot2, devtools, and WMWssp (R Foundation for Statistical Computing, Vienna, Austria), alpha was set at <0.05.

## 3. Results

All demographic, fitness, lifestyle behaviors, personality traits, and mood state variables are provided in Table 1. The majority (84%) of the sample met the ACFT standards for the PH. Although not statistically significant, the group that did not meet the ACFT PH standards self-reported greater durations of total PA (Pass: 762.82 ± 797.19, Fail: 835 ± 855.60), but the group who met the standards engaged in greater VPA (Pass: 261.25 ± 141.76, Fail: 135.00 ± 148.49, rg = 0.500). A large effect for VO_2max_ (*p* = 0.002, rg = 0.695) and plank hold duration (*p* < 0.001, rg = 0.948) were found to have significant group differences between those who passed and failed the PH determined by the ACFT standards (Table 1). PH duration had weak, negative correlations with fat mass (ρ = −0.38, *p* = 0.007) and BMI (ρ = −0.35, *p* = 0.013) (Table 2). For fitness parameters, PH duration was found to have a weak positive correlation with maximal push-ups (ρ = 0.29, *p* = 0.045) (Table 3). Additionally, PH duration had moderate, positive correlation with state physical (ρ = 0.61, *p* = 0.047) energy (Table 4). There were no significant correlations between PH duration and variables for grit, lifestyle behaviors, and personality traits. PH time was the only fitness variable for which males did not outperform females (*p* = 0.83, rg = 0.048).

## 4. Discussion

The main purpose of this exploratory study was to analyze how a maximum PH correlated with body composition, fitness, lifestyle behaviors, and mental and emotional health in TA. A secondary purpose was also to assess how those variables differ between those who pass and fail the event. The results of this exploratory study partially supported our hypothesis as it was found that PH time was negatively related to several measures of body composition (i.e., fat mass, BMI) and positively related to upper body muscular endurance (i.e., maximum push-ups). Additionally, we did find that those self-reporting greater state physical energy performed better on the PH. When we categorized participants into “Pass” and “Fail” groups based on the ACTF standards, VO_2max_ values were significantly different between the groups. Unexpectedly, there were no other significant differences in measures between the “Pass” and “Fail” groups. It is noteworthy that lifestyle variables and grit were not significantly associated with PH performance; however, our sample was small and rather homogenous in regard to many of these variables, which likely affected our findings.

### 4.1. Plank and Body Composition

Despite routine fitness testing, being overweight and obese is prevalent in the U.S. military, law enforcement, and firefighter populations [53,54]. Individuals classified as overweight or obese may display decreased athletic performance. Obese firefighters displayed 27% lower back and core endurance scores than their non-obese counterparts in a study by Mayer et al. [54]. In the current study, 82% of the sample were categorized as overweight and 10% were considered obese per BMI standards. Additionally, negative correlations were observed by Mayer et al. between BMI and body fat percent with core and back endurance [54]. Similarly, in the current study fat free mass and BMI were negatively correlated with PH outcomes. Two of the “Big 3” modifiable lifestyle behaviors, physical activity and diet, can directly contribute to BMI and fat free mass. Thus, individuals at risk for performing poorly or failing the PH due to poor body composition may benefit from a holistic approach rather than strict PH training.

### 4.2. Plank and Fitness Assessments

VO_2max_ was the only fitness assessment that was significantly different between PH pass/fail groups. These findings were not unexpected as core endurance training has been found to increase running economy, VO_2max_, and running performance in both fit and unfit populations [55,56]. Greater core endurance may lead to better running economy and efficiency which would contribute to better performance on aerobic fitness assessments. For example, in U.S. Army soldiers, PH performance has been found to be moderately correlated with time to complete a 3200 m march with a 25 kg load [11].

Push-ups were the only fitness assessment significantly correlated with PH performance. This was expected because push-ups require an individual to maintain their body in a straight line, such as a plank. Because proper push-up form requires core endurance, testing both in the same session may lead to increased fatigue on the latter event. However, due to the weak correlation, the two assessments may not be redundant and may not warrant the exclusion of one of the two from the ACFT using criteria similar to that of Cesario et al. [57]. Practitioners should provide adequate rest periods between the two assessments for their TA to reduce the risk of carry over fatigue.

### 4.3. Plank & Mobility/Balance

Greater core strength and endurance has been found to be correlated with a decreased risk of musculoskeletal injury in athletes and general population [12,58]. Likewise, YBT and FMS outcomes are indicators of injury risk in tactical athletes [59]. Thus, core endurance, YBT, and FMS outcomes are of importance to tactical strength and conditioning professionals. Previous literature found moderately-strong correlations between PH outcomes and single-leg balance in soldiers [10]. Similarly, a significant, weak correlation was found between PH and FMS scores in firefighters and between trunk flexor and extensor endurance and FMS scores in military personnel [60,61]. However, the current study found no significant relationship between a maximum PH and YBT outcomes in TA. It is possible no relationship was found in the current study because only the anterior portion of the YBT was tested. Additionally, we found no significant relationship between PH and outcomes of the FMS overhead squat and the Apley Scratch shoulder mobility test. Okada et al. also found no significant correlations between core stability and FMS scores [62]. Therefore, core endurance may contribute to injury risk in a different way than the components of the YBT and FMS, and thus, all three assessments may complement one another when ascertaining injury risk.

### 4.4. Plank & Health Behaviors

Despite assessing numerous lifestyle and health behaviors and moods, PH performance was only significantly correlated with state physical energy in this population of TA. In regard to lifestyle behaviors, this finding can be interpreted as living a healthy lifestyle does not by itself equate to greater levels of fitness and vice versa (i.e., being fitter does not mean one necessarily displays healthy lifestyle behaviors). Furthermore, it has been reported that state and trait physical and mental energy were indicators of postural control and gait [26]. The PH is an event that requires isometric control of a specific posture, thus, the physical energy of a TA on that specific testing day may be one of the greatest contributors to performance. Grit has been previously correlated with physical performance in cadets and active duty military [29,30]. It is plausible that because the TA in the current study scored similarly on levels of grit, the lack of variability resulted in non-significant findings. Previous research has used larger populations where even small differences in grit may be reflected in performance and positively influence engagement in healthy lifestyle behaviors [28,63].

### 4.5. Limitations & Future Research

The sample population was comprised of professional law enforcement officers (*n* = 29) and firefighters (*n* = 20). Anecdotally, numerous participants mentioned a history of prior military service; however, it was not formerly documented as part of the research procedures. In comparison, the Active Duty U.S. Army mean age is 27.0 years for enlisted soldiers and 34.7 years for officers with a male:female ratio of 83:17 [64]. However, the current sample population has body composition and fitness outcomes similar to that of an Active Duty 101st Airborne Division cohort [65]. Regardless of population, this is one of the first studies to analyze the PH’s correlation with body composition, fitness assessments, and health and lifestyle behaviors. Most research with TA correlated the PH with occupation specific tasks such marksmanship, balance, one-repetition maximum box lift, and ruck march performance [9,10,11,62]. Outside of that, a majority of research utilizing TA examines the sit-up event [66]. Therefore, comparisons and contrasts between results must also consider utilizing research on athletes and the general population.

A main limitation of the study was the modest sample size. Post-hoc power analysis indicated that for the anthropometric, movement, and fitness data, the correlations were adequately powered; however, the lifestyle, mood, and personality variables had low power (<0.80) due to the smaller sample size (*n* = 18). Another limitation was that the PH was included in battery of assessments that could have induced fatigue prior to the plank. However, the ACFT and other occupational physical fitness assessments also include several assessments, thus making the current results more generalizable to test batteries rather than stand-alone assessment. Once more ACFT data is available, future research should assess the PH event in soldiers and how it may correlate with other ACFT events, body composition, and health and lifestyle behaviors. Moreover, data used in this study is part of an ongoing project; a recent addition of subjective measure to the methodology lead to a small data pool in terms of self-reported measures. Small variance in the subjective measure independent variables is not ideal for analyses and likely affected our findings. Due to the nature of fitness assessments, it is plausible that participants did not give their best effort during testing. All participants were given the same instructions and similar levels of encouragement during testing to maximize the likelihood of best effort. However, because participation was voluntary and performance was not consequential to their jobs, unlike the ACFT, participants may not have given full effort. Lastly, due to time constraints, only two of the seven FMS assessments were given. Thus, a composite FMS score could not be given. Future research should administer the entire FMS and YBT tests and ascertain how they may relate to performance on the entire ACFT.

## 5. Conclusions

The US Army has recently adopted the maximum PH event as part of their annual fitness testing (i.e., ACFT) despite limited literature supporting its use to predict occupational performance or injury. Considering the variables associated with PH performance (i.e., body mass index, fat mass, push-ups, state physical energy), as well as the only variable within this study that was significantly different between PH pass and fail groups was VO_2max,_ there are several preliminary conclusions that can be provided from this exploratory study. First, while the PH event is considered a test of core endurance, not aerobic fitness, it seems the two fitness measures may be intertwined and those with poor aerobic fitness are more at risk for failing the event. Additionally, individuals with greater BMI and fat mass may display worse performance on the PH. The lack of difference in PH performance between males and females in the study provides support for the PH as a ‘gender neutral’ assessment. Thus, to improve PH performance, it would be suggested that strength and conditioning practitioners implement training to enhance VO_2max_ and body composition. In regard to PH assessment it is appears that a TA’s physical energy on a testing day may influence their PH performance. Factors such as the time of day testing occurs, testing after a shift, or testing groups of TA together could all influence physical energy [67,68]. These should be documented to aid in interpreting individual results over multiple tests on the same individual.

## Figures and Tables

**Table 1:** Demographic, fitness, lifestyle, and personality characteristics of the tactical athletes.

Variables	Total: Mean (SD)	Pass: Mean (SD)	Pass: (Min, Max)	Fail: Mean (SD)	Fail: (Min, Max)	Effect Size (rg)	*p*-Value
Demographics and Anthropometrics (Total: *n* = 49, Pass: *n* = 41, Fail: *n* = 8)							
Age	38.39 (7.78)	38.88 (7.46)	(23.00, 25.00)	35.00 (9.07)	(23.00, 50.00)	0.250	0.272
Years of Service	11.98 (8.31)	11.81 (8.07)	(0.00, 30.00)	11.44 (9.66)	(1.50, 26.00)	0.061	0.796
Height (cm)	176.35 (8.02)	175.53 (8.06)	(158.00, 194.50)	179.59 (7.56)	(168.00, 190.00)	0.259	0.256
Mass (kg)	84.42 (15.13)	82.10 (12.25)	(47.78, 102.60)	95.60 (23.65)	(61.70, 139.9)	0.409	0.072
Body Fat (%)	23.12 (7.03)	22.45 (6.69)	(7.50, 35.20)	26.69 (8.52)	(15.20, 40.8)	0.256	0.261
Fat Free Mass (kg)	65.95 (11.26)	64.47 (10.90)	(39.19, 92.50)	72.93 (11.75)	(51.88, 87.94)	0.384	0.091
Fat Mass (kg)	19.99 (8.24)	18.71 (6.10)	(5.10, 32.30)	26.53 (14.13)	(11.30, 57.00)	0.402	0.076
BMI	27.01 (3.83)	26.54 (2.96)	(18.50, 32.79)	29.48 (6.61)	(20.73, 43.66)	0.335	0.140
Movement (Total: *n* = 49, Pass: *n* = 41, Fail: *n* = 8)							
WSR (cm)	30.96 (9.57)	30.18 (9.25)	(14.5, 50.50)	35.19 (11.25)	(22.00, 58.00)	0.256	0.261
YBTA (cm)	3.52 (2.88)	3.61 (3.04)	(0.00, 13.00)	2.75 (1.91)	(1.00, 6.00)	0.155	0.495
Overhead Squat	1.67 (0.63)	1.73 (0.60)	(0.00, 3.00)	1.38 (0.74)	(0.00, 2.00)	0.256	0.187
SMR (cm)	25.67 (12.01)	25.04 (10.33)	(0.00, 53.00)	28.38 (19.54)	(0.00, 68.00)	0.009	0.978
SML (cm)	28.04 (12.53	27.70 (11.11)	(0.00, 55.00)	29.12 (19.61)	(0.00, 66.00)	0.012	0.967
Fitness (Total: *n* = 49, Pass: *n* = 41, Fail: *n* = 8)							
Vertical Jump (in)	21.38 (4.15)	21.47 (4.03)	(13.80, 29.60)	21.16 (5.19)	(15.00, 28.70)	0.064	0.786
1RM BP (kg)	98.11 (26.95)	98.84 (25.85)	(20.41, 145.45)	92.27 (34.24)	(38.50, 127.01)	0.092	0.686
Pull up (reps)	8.57 (6.02)	9.07 (5.92)	(0.00, 21.00)	5.88 (6.58)	(0.00, 15.00)	0.290	0.202
Push up (reps)	38.06 (19.17)	39.50 (18.66)	(5.00, 105.00)	29.38 (21.49)	(6.00, 60.00)	0.271	0.233
Plank Hold (s)	109.21 (42.99)	115.19 (35.28)	(72.00, 249.00)	61.80 (13.09)	(36.00, 78.00)	0.948	<0.001 ***
VO_2max_ (mL·kg^−1^·min^−1^)	44.71 (5.11)	45.78 (4.75)	(35.90, 58.50)	39.48 (3.92)	(34.2, 44.09)	0.695	0.002 **
Lifestyle Behaviors (Total: *n* = 18, Pass: *n* = 16, Fail: *n* = 2)							
Grit	3.87 (0.46)	3.84 (0.48)	(3.00, 5.00)	4.06 (0.27)	(3.87, 4.25)	0.344	0.478
REAPS	29.39 (3.15)	29.25 (3.28)	(25.00, 35.00)	30.50 (2.12)	(29.00, 32.00)	0.250	0.620
PSQI	4.83 (1.86)	4.94 (1.95)	(3.00, 11.00)	4.00 (0.00)	(4.00, 4.00)	0.312	0.498
VPA (min/wk)	247.22 (143.86)	261.25 (141.76)	(40.00, 540.00)	135.00 (148.49)	(30.00, 240.00)	0.500	0.289
MPA (min/wk)	202.78 (219.73)	194.38 (230.13)	(0.00, 720.00)	270.00 (127.28)	(180.00, 360.00)	0.406	0.397
LPA (min/wk)	320.83 (425.39)	307.19 (425.30)	(0.00, 1680.00)	430.00 (579.83)	(20.00, 840.00)	0.031	1.000
Sitting (min/wk)	1908.33 (2455.85)	2041.88 (2581.50)	(240.00, 10,500.00)	840.00 (0.00)	(840.00, 840.00)	0.063	0.944
Moods and Personality (Total: *n* = 18, Pass: *n* = 16, Fail: *n* = 2)							
Trait PE	7.44 (2.71)	7.56 (2.80)	(1.00, 12.00)	6.50 (2.12)	(5.00, 8.00)	0.344	0.476
Trait PF	3.61 (2.06)	3.31 (1.85)	(0.00, 6.00)	6.00 (2.83)	(4.00, 8.00)	0.656	0.154
Trait ME	7.67 (2.25)	7.75 (2.35)	(3.00, 12.00)	7.00 (1.41)	(6.00, 8.00)	0.219	0.667
Trait MF	2.94 (1.98)	2.88 (2.09)	(0.00, 6.00)	3.50 (0.71)	(3.00, 4.00)	0.281	0.554
State PE	201.28 (62.43)	205.00 (64.73)	(90.00, 300.00)	171.50 (37.48)	(145.00, 198.00)	0.375	0.439
State PF	78.39 (63.86)	76.75 (61.77)	(3.00, 220.00)	91.50 (108.19)	(15.00, 168.00)	0.063	0.944
State ME	207.06 (61.80)	208.25 (65.63)	(99.00, 300.00)	197.50 (10.61)	(190.00, 205.00)	0.000	1.000
State MF	69.00 (66.54)	67.88 (66.24)	(0.00, 253.00)	78.00 (96.17)	(10.00, 146.00)	0.063	0.944

Note: ** *p* ≤ 0.01; *** *p* ≤ 0.001; Abbreviations: 1RM—1 Repetition Maximum, BP—Bench Press BMI—Body Mass Index, WSR = Wall Sit and Reach, YBTA = Y-balance Test Asymmetry, SMR = Shoulder Mobility Right, SML—Shoulder Mobility Left, VO_2max_—Maximal Oxygen Consumption, REAPS—Rapid Eating Assessment for Participants, PSQI—Pittsburg Sleep Quality Index, VPA—vigorous physical activity, MPA—moderate physical activity, LPA—light physical activity, PE—Physical Energy, PF—Physical Fatigue, ME—Mental Energy, MF—Mental fatigue.

**Table 2:** Correlation Matrix—Demographics, Body Composition, and Mobility.

	Plank	Age	YOS	Height	Mass	BF	FFM	FM	BMI	WSR	YBTA	Squat	SMA
SMA	0.061	−0.023	−0.147	−0.101	−0.034	0.081	−0.139	0.073	0.015	−0.254	0.128	0.287 *	-
Squat	0.277	−0.064	0.049	0.059	−0.047	−0.178	−0.061	−0.190	−0.168	−0.029	0.000	-	
YBTA	0.190	0.128	0.019	−0.046	0.014	−0.030	0.056	−0.017	0.047	−0.001	-		
WSR	−0.073	−0.430 **	−0.225	−0.021	−0.091	−0.031	−0.037	−0.040	−0.121	-			
BMI	−0.351 *	0.180	0.152	0.193	0.776 **	0.478 **	0.369 **	0.747 **	-				
FM	−0.379 **	0.146	0.073	0.132	0.569 **	0.882 **	−0.019	-					
FFM	−0.246	0.051	0.153	0.650 **	0.645 **	−0.374 **	-						
BF	−0.267	0.107	0.020	−0.198	0.191	-							
Mass	−0.270	0.150	0.213	0.729 **	-								
Height	−0.072	0.055	0.121	-									
YOS	0.102	0.748 **	-										
Age	0.207	-											
Plank	-												

Statistical significance: * *p* ≤ 0.05, ** *p* ≤ 0.01. Abbreviations: YOS—Years of Service, BF—Body Fat %, FFM—Fat Free Mass, FM—Fat Mass, BMI—Body Mass Index, WSR—Wall Sit and Reach, YBTA—Y-Balance Test Asymmetries, SMA—Shoulder Mobility Asymmetry.

**Table 3:** Correlation Matrix—Performance.

	Plank	CMJ	Bench	Pull-Up	Push-Up	VO_2max_
VO_2max_	0.277	0.223	0.043	0.300 *	0.142	-
Push	0.287 *	0.417	0.537 *	0.724 **	-	
Pull	0.139	0.613 **	0.499	-		
Bench	0.073	0.410	-			
CMJ	−0.026	-				
Plank	-					

Statistical significance: * *p* ≤ 0.05, ** *p* ≤ 0.01. Abbreviations: CMJ—Countermovement Jump, VO_2max_—Maximum Oxygen Consumption.

**Table 4:** Correlation Matrix—Mood States.

	SPE	SPF	SME	SMF	Grit	REAPS	PSQI	Plank
Plank	0.609 *	0.046	0.573	−0.109	−0.166	0.027	−0.369	-
PSQI	−0.431	0.497	−0.666 *	−0.532	0.107	0.111	-	
REAPS	−0.009	−0.025	−0.581	0.380	0.192	-		
Grit	−0.285	−0.297	−0.386	0.115	-			
SMF	−0.591	0.601 *	−0.491	-				
SME	0.555	0.005	-					
SPF	−0.228	-						
SPE	-							

Statistical significance: * *p* ≤ 0.05. Abbreviations: SPE—State Physical Energy, SPF = State Physical Fatigue, SME—State Mental Energy, SMF—State Mental Fatigue, REAPS—Rapid Eating Assessment for Participants Short Version, PSQI—Pittsburgh Sleep Quality Index.

## Data Availability

The data and code that support the findings of this study are available from the corresponding author, M.S.v.d.W., upon reasonable request.
